# Validity of a Self-administered Food Frequency Questionnaire Used in the 5-year Follow-up Survey of the JPHC Study to Assess Selenium Intake: Comparison with Dietary Records and Blood Levels

**DOI:** 10.2188/jea.13.1sup_92

**Published:** 2007-11-30

**Authors:** Kanae Karita, Satoshi Sasaki, Junko Ishihara, Shoichiro Tsugane

**Affiliations:** 1 Department of Hygiene and Public Health, Teikyo University School of Medicine.; 2 Epidemiology and Biostatistics Division, National Cancer Center Research Institute East.; 3 Cancer Information and Epidemiology Division, National Cancer Center Research Institute.

**Keywords:** selenium, serum, erythrocytes, validity, dietary records, food frequency questionnaire

## Abstract

Selenium (Se) levels in serum and erythrocytes were measured in 215 adults to assess the relative validity of Se intake estimated from 28-day dietary records (DR) and the food frequency questionnaire (FFQ) used in the 5-year follow-up survey of the JPHC study. Se intake estimated from DR was correlated with that from the FFQ both in males and females (r=0.36 and r=0.32, respectively). Se levels in erythrocytes were weakly correlated with Se intake by DR for men (r=0.21) after adjustments for the total energy intake, though no significant correlation was found for women nor in crude values for either sex. Although Se intake estimated from our FFQ was correlated with that from the DR, no correlation was observed between the Se level in serum and estimated Se intake. In this population, the serum Se level was not a good biomarker for estimated Se intake.

Selenium (Se) is an essential nutrient for which new roles are continually being recognized. Experimental studies indicate that Se possesses anticarcinogenic properties.^1^^,^^2^ It acts as a cofactor in the glutathione peroxidase (GSH-Px) of cell membranes and is important in cellular deintoxication of peroxides and free radicals.^2^^,^^3^

In humans, epidemiological correlation studies have noted that regions with small amounts of Se in foods have high cancer mortality rates,^4^^,^^5^ and low serum Se associates with an increased risk of cardiovascular disease.^6^ Recent case-control studies used stored sera that were obtained before cancer diagnoses for Se analysis.^7^ However, there were insufficient numbers of cancer cases by specific sites to determine clearly which cancers were affected by low Se.

Geographic comparisons of blood Se levels have demonstrated distinct variations in Se status, which roughly correlates with the dietary Se intake.^8^ A review of human blood Se levels revealed large differences in the apparent dietary status of individuals in different areas of the world. Among Japanese, the nutritional status of Se is reported to be sufficient.^9^^,^^10^ The question arises as to whether blood Se measurement is a reliable index of dietary Se intake in Japanese. The two-fold purpose of this study was to assess the relationship between dietary intake of Se and Se levels in serum and erythrocytes in a community-dwelling sample of Japanese adults, and to examine the validity of a self-administered FFQ used in the JPHC study.

## METHODS

### Estimation of Se Intake

As previously described,^11^ 7-day dietary records (DR) were provided a total of four times, once in each season of the year in the PHC areas of Ninohe (Iwate), Yokote (Akita) and Saku (Nagano). In Ishikawa (Okinawa), they were collected only in winter and summer. In this analysis, 14-day records (DR) in Ishikawa and 28-day records (DR) in the other 3 areas were included. The subjects were requested to fill in the FFQ three months after they completed their DRs. Se intake was calculated using the Table of Trace Elements Contents in Japanese Foodstuffs^12^ and the analyzed values.^13^ When some foodstuff data of Se were missing, the values of foods regarded to be nutritionally comparable were used instead. The Se values in the composition table used for the calculation of intake from DR are available on a written request basis. Detailed calculation from FFQ and DR, performed with an ad-hoc computer program based on the classification of the Japanese Standard Food Composition Tables, was described elsewhere.^14^^,^^15^ Possible correlations of dietary Se intake ascertained in 102 males and 113 females were examined.

### Analyses of Blood Se Levels

Blood was obtained by venipuncture just before the winter and summer DR surveys. After blood was separated into erythrocytes and serum by centrifugation, all the samples for blood Se measurements were stored in trace element-free tubes in a deep freezer until analyzed. The serum concentrations of Se and erythrocytes were measured by the Watkinson's method^16^ using fluorometry (Hitachi F-3010) after ashing with HNO_3_ and HClO_4_. Quality control was simultaneously assessed by measuring the reference materials (IAEA; Animal Blood (A-13), NIST; Bovine Liver 1577a). The obtained results were confirmed to be within the certified values of the reference materials. The sensitivity of this method in our laboratory was 1 ng of Se, and the detection limit (defined as three times the SD of the analytical blank) was 0.01 *μ*mol/l serum and 0.01 nmol/g erythrocytes. Analysis of duplicate specimens gave a coefficient of variation less than 5%. A total of 180 subjects (85 males and 95 females) were included in the analysis of Se in serum and erythrocytes.

### Statistical Analysis

The estimated intake for dietary Se and blood levels of Se were calculated by areas, seasons and sex and presented as mean, SD and median. The area differences in means were compared using one-way analysis of variance (ANOVA). Pearson and Spearman correlation coefficients were used to compare the two methods for dietary assessment of Se. Spearman rank correlation coefficients were computed for the Se serum level and erythrocytes vs. Se intake from FFQ and from DR. To remove covariation due to total energy intake, the correlation coefficients adjusted for energy intake were also calculated using residual regression models.^17^ All the statistical analyses were performed with a PC-SAS software package.

## RESULTS

The mean intake of Se estimated by the two methods was shown in Table 1. The mean, standard deviation and median for Se intake estimated by DR and FFQ were calculated by area and sex. Mean daily intake of 28-day DRs (14 days for Okinawa) was highest in Ninohe for men, and in Ninohe and Yokote for women. The mean intake was lowest in Ishikawa for both men and women. The mean intake of Se estimated by DR was significantly different among the 4 areas by ANOVA. Mean intake from FFQ was highest in Ninohe for men and in Saku for women. The mean intake from FFQ was lowest in Ishikawa for both men and women. The mean intake of Se estimated by FFQ was significantly different among the 4 areas by ANOVA. Daily Se intakes from DR were significantly higher than those estimated from FFQ for men in all areas, and for women in the Yokote and Ishikawa PHC areas (t-test p<0.05). 

**Table 1.** Selenium intake (*μ*g/day) assessed with DR and FFQ by area

	Ninohe PHC		Yokote PHC		Saku PHC		Ishikawa PHC		ANOVA p-value
			
	Mean±SD	Median	Mean±SD	Median	Mean±SD	Median	Mean±SD	Median
DR^1^									
Men (n=102)	n=24		n=28		n=23		n=27		<0.001
	200±36	208	188±37	186	191±40	191	155±43	152	
Women (n=113)	n=27		n=30		n=28		n=28		<0.001
	158±31	158	158±29	156	148±30		127±37	123	
FFQ									
Men (n=102)	n=24		n=28		n=23		n=27		<0.001
	162±84	146	125±48	110	151±56	151	96±46	87	
Women (n=113)	n=27		n=30		n=28		n=28		0.002
	132±68	104	113±44	107	153±108	133	83±29	75	

^1^Mean intakes from 28-day DR in Ninohe, Yokote and Saku PHC, and mean intake of 14-day DR in Ishikawa PHC.

Table 2 summarizes the correlations between the average intake of Se estimated by DR and those by FFQ for both crude and energy-adjusted values. The mean intake from DR was higher than that from FFQ for both men and women. Correlation coefficients between DR and FFQ were 0.36 and 0.32 for crude intake, and 0.34 and 0.26 for energy-adjusted intake, in men and women, respectively.

**Table 2.** Mean selenium intake (*μ*g/day) assessed with DR and FFQ in 4 areas, and Spearman correlation coefficients

	DR				FFQ				% Difference		
		
	Mean	±	SD	Median	Mean	±	SD	Median		Crude	Energy-^2^ adjusted
Men (n=102)	184	±	48	175	132	±	63	116	-28	0.36	0.34
Women (n=113)	146	±	37	142	120	±	72	101	-18	0.32	0.26

^1^(FFQ mean - DR mean)/DR mean (%).

^2^Energy intake was adjusted by residual model.

For n=102, r>0.20 = p<0.05, r>0.26 = p<0.01, r>0.33 = p<0.001.

For n=113, r>0.19 = p<0.05, r>0.25 = p<0.01, r>0.31 = p<0.001.

Table 3 shows Se levels in serum and erythrocytes by area, sex and season. Lower means of Se in erythrocytes were observed both in men and women of the Saku PHC area, while no apparent area difference was observed for serum Se levels.

**Table 3.** Mean±SD and median of selenium levels in serum (S-Se; *μ*mol/L) and erythrocytes (E-Se; nmol/g) by areas, seasons and sex

	Ninohe PHC		Yokote PHC		Saku PHC		Ishikawa PHC		ANOVA p-value
			
	Mean±SD	Median	Mean±SD	Median	Mean±SD	Median	Mean±SD	Median
Men (n)	22		24		19		20		
Winter									
S-Se	1.72±0.38	1.59	1.72±0.22	1.66	1.52±0.20	1.52	1.84±0.41	1.81	0.02
E-Se	3.60±0.73	3.35	4.12±0.85	3.97	3.52±0.53	3.47	4.54±0.69	4.53	<0.001
Summer									
S-Se	1.65±0.27	1.58	1.55±0.32	1.48	1.75±0.32	1.67	1.73±0.22	1.73	0.103
E-Se	4.63±0.57	4.63	3.80±0.94	3.81	3.66±0.64	3.53	4.41±1.07	4.35	<0.001
Mean of winter and summer									
S-Se	1.69±0.31	1.57	1.64±0.23	1.57	1.63±0.19	1.63	1.79±0.27	1.80	0.22
E-Se	4.11±0.59	4.02	3.96±0.71	3.87	3.59±0.47	3.61	4.47±0.75	4.42	<0.001

Women (n)	25		26		23		21		
Winter									
S-Se	1.61±0.16	1.65	1.64±0.28	1.59	1.74±0.24	1.73	1.65±0.16	1.62	0.173
E-Se	3.58±0.60	3.61	3.48±0.32	3.52	3.41±0.46	3.43	4.32±0.46	4.33	<0.001
Summer									
S-Se	1.54±0.14	1.51	1.41±0.29	1.36	1.51±0.23	1.48	1.67±0.16	1.62	0.002
E-Se	3.75±0.56	3.65	3.89±0.43	3.81	3.32±0.47	3.29	3.91±0.53	3.99	<0.001
Mean of winter and summer									
S-Se	1.57±0.14	1.57	1.52±0.27	1.50	1.63±0.20	1.58	1.66±0.15	1.62	0.109
E-Se	3.67±0.55	3.65	3.68±0.33	3.64	3.36±0.45	3.30	4.12±0.41	4.06	<0.001

Correlations between dietary Se intake and blood level of Se are shown in Table 4. There were no significant correlations between blood levels of Se and DR estimations in crude values (r=-0.07 and -0.05 for S-Se, and r=0.03 and -0.11 for E-Se, in men and women, respectively). When Se intake was adjusted for energy by the residual model, the correlation coefficients for E-Se improved to 0.21 in men. There were no significant correlations between blood levels of Se and FFQ estimations in crude values (r=-0.05 and -0.04 for S-Se, and r=-0.03 and -0.17 for E-Se, in men and women, respectively). The correlation coefficients improved slightly, but not significantly after energy adjustment.

**Table 4.** Spearman correlation coefficients between DR, FFQ and biomarkers of Se (average of winter and summer)

		Blood Se^1^	Correlation with DR		Correlation with DR	
			
	n	Mean±SD	Crude	Energy-adjusted	Crude	Energy-adjusted
Men		1.68±0.26	-0.07	0.01	-0.05	0.16
S-Se	85	4.03±0.69	0.03	0.21	-0.03	0.15
E-Se	85					

Women						
S-Se	95	1.59±0.20	-0.05	-0.04	-0.04	-0.02
E-Se	95	3.71±0.51	-0.11	0.11	-0.17	0.13

^1^S-Se: selenium levels in serum (*μ*mol/L); E-Se: selenium levels in erythrocytes (nmol/g).

For n=85, r>0.21 = p<0.05, r>0.28 = p<0.01, r>0.35 = p<0.001.

For n=95, r>0.21 = p<0.05, r>0.27 = p<0.01, r>0.34 = p<0.001.

Table 5 shows the cumulative % contribution of the top 20 foods for selenium assessed by DR. The top 20 foods contributed 53% of total selenium intake in both sexes. Chicken eggs were the greatest contributor, followed by rice, which was second in men and third in women. Several types of fish products were also important sources of Se. Fourteen among the top 20 foods were fish products. Some, 32% and 30% of the total Se was derived from fish in men and women, respectively.

**Table 5.** Cumulative % contribution of the top 20 foods for selenium assessed by DR

Food code^1^	Description^1^	*μ*g/day	Cumulative^2^ percent
Men (n=102)			
10-5a	Chicken egg/Whole egg/fresh	17.1	9.3
1-41d	Rice (Paddy rice)/Grains/Well-milled rice	10.7	15.2
8-150a	Tuna/Bluefin tuna,raw/Lean meat	10.2	20.7
8-31	Sardines/Boiled and dried	9.7	26.0
8-78a	Mild salted chum salmon/Uncooked	4.9	28.7
8-153c	Tuna/Canned with/Oil; solids and liquid	4.9	31.4
8-206a	Squid and cuttlefish/Raw/Uncooked	4.7	34.0
8-81	Salmon/Salted roe	4.1	36.2
8-50	Skipjack and frigate mackerel/Skipjack, raw	3.8	38.3
1-26a	Chinese noodle i.e., Lao Mien/Raw, dry form	3.4	40.1
11-2	Liquid milk/Ordinary liquid milk	3.3	41.9
8-250	Fish paste products/Yaki-chikuwa, broiled	2.7	43.4
8-95a	Pacific saury/Raw/Uncooked	2.7	44.9
1-21b	Japanese noodles/Udon/Boiled	2.5	46.2
8-109	Sea breams/Crimson sea bream, raw	2.5	47.6
8-65	Kichiji, rockfish/Raw	2.0	48.7
8-244	Sea squirt/Raw	2.0	49.8
8-246	Fish paste products/Mushi-kamaboko	2.0	50.8
8-4a	Japanese horse mackerel, raw/Uncooked	1.9	51.9
9-70a	Pork and/Belly without separable fat	1.9	52.9
Women (n=113)			
10-5a	Chicken egg/Whole egg/fresh	14.3	9.7
8-31	Sardines/Boiled and dried	8.6	15.4
1-41d	Rice (Paddy rice)/Grains/Well-milled rice	6.9	20.1
8-150a	Tuna/Bluefin tuna, raw/Lean meat	6.4	24.4
8-78a	Mild salted chum salmon/Uncooked	4.5	27.4
8-153c	Tuna/Canned with Oil; solids and liquid	4.3	30.4
11-2	Liquid milk/Ordinary liquid milk	3.7	32.9
8-81	Salmon/Salted roe	3.5	35.2
8-206a	Squid and cuttlefish/Raw/Uncooked	3.3	37.5
1-26a	Chinese noodle i.e., Lao Mien/Raw, dry form	2.7	39.3
8-250	Fish paste products/Yaki-chikuwa, broiled	2.7	41.1
8-50	Skipjack and frigate mackerel/Skipjack, raw	2.6	42.9
8-95a	Pacific saury/Raw/Uncooked	2.2	44.4
1-21b	Japanese noodles/Udon/Boiled	2.2	45.9
9-70a	Pork and/Belly without separable fat	1.8	47.1
8-109	Sea breams/Crimson sea bream, raw	1.7	48.2
8-246	Fish paste products/Mushi-kamaboko	1.7	49.4
8-65	Kichiji, rockfish/Raw	1.6	50.5
8-54	Skipjack/Katsuo-bushi, dried strip	1.5	51.5
9-68a	Pork and separable fat/Loin, separable lean	1.4	52.5

^1^Food codes and descriptions correspond to those of the Standard Tables of Food Composition, 4th revised edition in Japan by Science and Technology Agency.

^2^Data on subjects in Ishikawa PHC (14-day data) were counted twice for 28-day data.

## DISCUSSION

Despite considerable interest in the anticarcinogenic effects of Se, its estimated dietary intake has not been fully investigated for its validity in a Japanese population. In the present study, regional differences were found in the trend in Se intake. Mean Se intake was estimated to be about 120-200 *μ*g/day/person by DR. WHO officially released an updated trace element nutrition report including Se in 1996.^18^ According to this report, the population minimum mean intake to meet normative requirements for adult males was 40 *μ*g of Se/day, which was calculated from the amount needed to achieve two-thirds of the maximal plasma GSH-Px activity. Our results indicate that mean Se intake in Japanese was 3 to 5 times higher than this minimum requirement, consistent with the previously reported values for dietary Se intake in Japanese.^10^^,^^19^

The correlation coefficients we obtained for Se intake between DR and FFQ were not so high, i.e., r=0.26-0.36. Dietary Se intake estimated from FFQ was significantly lower than that estimated from DR. The Japanese diet is characterized by a large number of food items and seasonal variations, so inevitable errors such as omission of minor but Se-rich foods from the food frequency questionnaires could have resulted in underestimation of Se intake. We modified the food composition table of Se for use with Japanese populations in general, and arbitrarily substituted for the missing values of the minor but Se-rich foods. Thus, the quality of the table must be improved by further analyses of Se in duplicate food collection from Japanese samples.

The Se level in a single blood specimen provided only a rough and insufficient estimate of intake for each subject because associations between intake of nutrients and blood nutrient status can not be considered primary effects of diet if they are the result of differences in body size and metabolic efficiency. Generally, even vitamins and minerals are positively correlated with total energy intake, because heavier and less metabolically efficient persons tend to eat more of everything. The subjects with higher energy intakes consume larger portions, rather than more food items, so nutrient intakes may be innately adjusted for total energy intake. Energy adjustment also reduces inter-individual variation due to over- or underreporting of intake. In the present study, significant correlations were not observed between estimated Se intake and the blood level of Se; however, when the energy residual model was adapted, the adjusted intake from DR showed positive correlations with erythrocyte levels of Se. The adjusted biomarkers are more clearly related to the assessed intake, which was indicated in some nutrients including minerals with the energy-adjusted intake among Japanese populations.^20^^,^^21^

None of the estimated Se intake was related to serum Se levels in the present study. A few studies have compared serum Se values to the dietary intake of Se,^22^^,^^23^ and demonstrated a positive correlation between the two variables when the Se intake per day was estimated from subjects with low Se serum levels. It is difficult to detect the relation between Se levels in serum or plasma and estimated dietary intake where the nutritional status of Se is considered sufficient. Though there is no accepted gold standard for assessing individual amounts of dietary Se intake, erythrocyte Se can be a more reliable biomarker, because its biological half-life is longer and considered to be a long-term indicator of dietary intake compared to serum levels. Serum Se measurement, on the other hand, only reflects the latest dietary intake of Se. Moreover, Se present in blood is dependent on the quantity and manner in which Se is ingested in the daily diet. For example, meat and fish are a reliable source of Se, but the bioavailability of Se from these foods is reportedly rather low.^24^ Se contents in plants are affected by the levels and the availability of the soil in which they are grown.^25^^,^^26^ Additional research is needed to identify which foods are the major contributors of Se in erythrocytes or serum, in order to validate the dietary methods more conclusively.

Fish was the most important food source of Se, followed by eggs. Similar results have been reported in a study of middle-aged Japanese men.^27^ However, the total intake of Se, estimated here by DR to be 184 and 146 *μ*g/day in men and women, respectively, was considerably higher than the 127 *μ*g/day^27^ reported in the men in the above study. The reason for this difference remains unclear in terms of either the difference in population characteristics or the difference in the food composition table used in each study. Further refinement of the food composition table seems necessary to estimate Se intake levels of Japanese populations more accurately, and to use the estimated intake assessed with DR as the gold standard in validation studies of newly-developed dietary assessment methods.

In conclusion, we compared blood Se values to the dietary intake of Se to determine the validity of our dietary survey methods. Although Se intake estimated from our FFQ was correlated with that from the DRs, most of the mean Se intakes thus estimated were lower than those estimated from DRs. No significant correlations were found between blood Se levels and estimated dietary Se intake in crude values, yet the validity was improved with erythrocyte levels of Se after employing Se intake adjusted for energy intake. Given the adequate status of Se among Japanese, the validity level of our nutritional survey can be regarded reasonable, as indicated by the positive correlations between energy-adjusted intake and the erythrocyte level of Se.
